# *N*-Benzyl-*N*-cyclo­hexyl­benzene­sulfonamide

**DOI:** 10.1107/S1600536809046960

**Published:** 2009-11-11

**Authors:** Zeeshan Haider, Islam Ullah Khan, Muhammad Zia-ur-Rehman, Muhammad Nadeem Arshad

**Affiliations:** a Department of Chemistry, Government College University, Lahore 54000, Pakistan; b Applied Chemistry Research Centre, PCSIR Laboratories Complex, Ferozpure Road, Lahore 54600, Pakistan

## Abstract

In the title compound, C_19_H_23_NO_2_S, the cyclo­hexyl ring exists in a chair form. The dihedral angle between the two terminal phenyl rings is 86.70 (6)°. No significant inter­actions are observed except for a weak intra­molecular C—H⋯O hydrogen bond.

## Related literature

For the biological activity of sulfonamides, see: Innocenti *et al.* (2004 ▶); Ozbek *et al.* (2007 ▶); Parari *et al.* (2008 ▶); Ratish *et al.* (2009 ▶); Selnam *et al.* (2001 ▶); For related structures, see: Khan *et al.* (2009 ▶); Zia-ur-Rehman *et al.* (2009 ▶) Gowda *et al.* (2007*a* ▶,*b* ▶,*c* ▶). For bond length data, see: Allen *et al.* (1987 ▶).
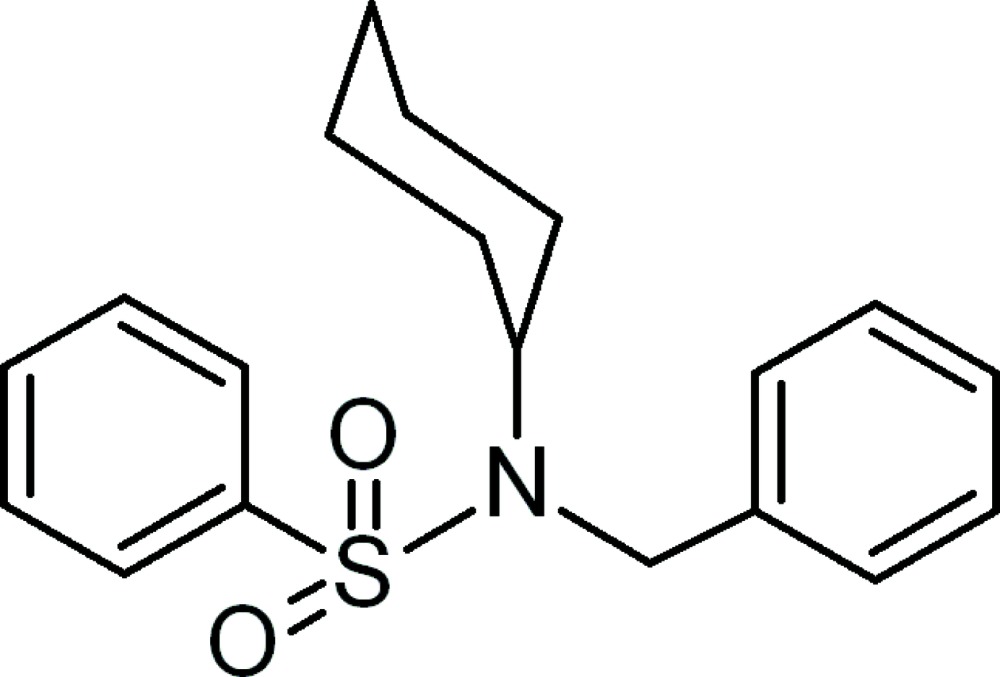


## Experimental

### 

#### Crystal data

C_19_H_23_NO_2_S
*M* *_r_* = 329.44
Orthorhombic, 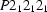

*a* = 9.1996 (5) Å
*b* = 11.0406 (5) Å
*c* = 17.1897 (9) Å
*V* = 1745.94 (15) Å^3^
*Z* = 4
Mo *K*α radiation
μ = 0.20 mm^−1^
*T* = 296 K
0.39 × 0.34 × 0.28 mm

#### Data collection

Bruker APEXII CCD area-detector diffractometer
Absorption correction: multi-scan (**SADABS**; Sheldrick, 1996 ▶) *T*_min_ = 0.928, *T*_max_ = 0.948
10897 measured reflections
4306 independent reflections
3507 reflections with *I* > 2σ(*I*)
*R*_int_ = 0.023

#### Refinement

*R*[*F* ^2^ > 2σ(*F* ^2^)] = 0.037
*wR*(*F* ^2^) = 0.094
*S* = 1.03
4306 reflections
208 parameters
H-atom parameters constrained
Δρ_max_ = 0.24 e Å^−3^
Δρ_min_ = −0.24 e Å^−3^
Absolute structure: Flack (1983 ▶), 1853 Friedel pairs
Flack parameter: 0.03 (7)

### 

Data collection: *APEX2* (Bruker, 2007 ▶); cell refinement: *SAINT* (Bruker, 2007 ▶); data reduction: *SAINT*; program(s) used to solve structure: *SHELXS97* (Sheldrick, 2008 ▶); program(s) used to refine structure: *SHELXL97* (Sheldrick, 2008 ▶); molecular graphics: *PLATON* (Spek, 2009 ▶) and *Mercury* (Macrae *et al.*, 2006 ▶); software used to prepare material for publication: *SHELXTL* (Sheldrick, 2008 ▶) and local programs.

## Supplementary Material

Crystal structure: contains datablocks I, global. DOI: 10.1107/S1600536809046960/is2484sup1.cif

Structure factors: contains datablocks I. DOI: 10.1107/S1600536809046960/is2484Isup2.hkl

Additional supplementary materials:  crystallographic information; 3D view; checkCIF report

## Figures and Tables

**Table 1:** Hydrogen-bond geometry (Å, °)

*D*—H⋯*A*	*D*—H	H⋯*A*	*D*⋯*A*	*D*—H⋯*A*
C7—H7⋯O2	0.98	2.34	2.874 (2)	113

**Fig. 1.**
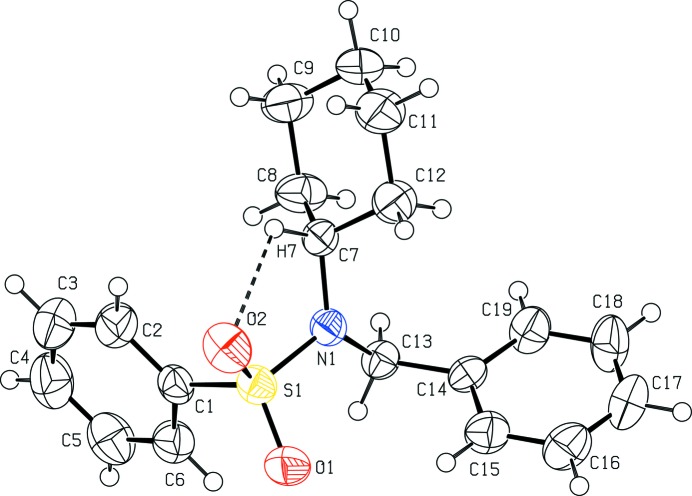
The molecular structure of (I), with displacement ellipsoids at the 50% probability level.

## supplementary crystallographic information

### Comment

Sulfonamides are familiar as biologically active compounds and possess anti-microbial (Ozbek *et al.*, 2007; Parari *et al.*, 2008), anti-inflamatory (Ratish *et al.*, 2009), anti HIV (Selnam *et al.*, 2001) and carbonic anhydrase inhibiton activity (Innocenti *et al.*, 2004). In the present paper, the structure of *N*-cyclohexyl-*N*-propyl benzene sulfonamide has been determined as part of a research program involving the synthesis of various sulfur containing heterocycles (Zia-ur-Rehman *et al.*, 2009; Khan *et al.*, 2009).

In the molecule of (I) (Fig. 1), bond lengths and bond angles are almost similar to those in the related molecules (Gowda *et al.*, 2007*a*,*b*,*c*) and are within normal ranges (Allen *et al.*, 1987). The benzene rings are essentially planar while cyclohexane ring is in the chair form. The dihedral angles between the phenyl (C1–C6) & benzyl ring (C14–C19), the phenyl (C1–C6) ring & the mean plane of cyclohexyl ring (C7–C12), and the benzyl (C14–C19) ring & the mean plane cyclohexyl ring (C7–C12) are 86.70 (6), 42.43 (8) and 55.47 (8)°, respectively, while the r.m.s. deviation for the phenyl (C1–C6), benzyl (C14–C19) and cyclohexyl (C7–C12) rings are 0.0021, 0.0036 and 0.2365 Å, respectively. An intramolecular C—H···O hydrogen bond gives rise to a five-membered hydrogen bonded ring (Table 1).

### Experimental

A mixture of *N*-cyclohexylbenzene sulfonamide (1 g, 0.43 mmol), sodium hydride (0.21 g, 0.88 mmol) and *N*, *N*-dimethylformamide (10 ml) was stirred at room temperature for half an hour followed by addition of benzyl chloride (0.114 g, 0.90 mmol). Stirring was continued further for a period of three hours and the contents were poured over crushed ice. Precipitated product was isolated, washed and crystallized from methanol-water mixture (50:50).

### Refinement

All H atoms were identified in a difference map and then were treated as riding (C—H = 0.93 or 0.97 Å), with *U*_iso_(H) = 1.2*U*_eq_(C).

### Crystal data

**Table:**

C_19_H_23_NO_2_S	*F*(000) = 704
*M**_r_* = 329.44	*D*_x_ = 1.253 Mg m^−^^3^
Orthorhombic, *P*2_1_2_1_2_1_	Mo *K*α radiation, λ = 0.71073 Å
Hall symbol: P 2ac 2ab	Cell parameters from 3979 reflections
*a* = 9.1996 (5) Å	θ = 2.3–21.8°
*b* = 11.0406 (5) Å	µ = 0.20 mm^−^^1^
*c* = 17.1897 (9) Å	*T* = 296 K
*V* = 1745.94 (15) Å^3^	Blocks, colourless
*Z* = 4	0.39 × 0.34 × 0.28 mm

### Data collection

**Table:**

Bruker APEXII CCD area-detector diffractometer	4306 independent reflections
Radiation source: fine-focus sealed tube	3507 reflections with *I* > 2σ(*I*)
graphite	*R*_int_ = 0.023
φ and ω scans	θ_max_ = 28.3°, θ_min_ = 2.2°
Absorption correction: multi-scan (*SADABS*; Sheldrick, 1996)	*h* = −12→11
*T*_min_ = 0.928, *T*_max_ = 0.948	*k* = −11→14
10897 measured reflections	*l* = −22→17

### Refinement

**Table:**

Refinement on *F*^2^	Secondary atom site location: difference Fourier map
Least-squares matrix: full	Hydrogen site location: inferred from neighbouring sites
*R*[*F*^2^ > 2σ(*F*^2^)] = 0.037	H-atom parameters constrained
*wR*(*F*^2^) = 0.094	*w* = 1/[σ^2^(*F*_o_^2^) + (0.049*P*)^2^ + 0.1094*P*] where *P* = (*F*_o_^2^ + 2*F*_c_^2^)/3
*S* = 1.03	(Δ/σ)_max_ < 0.001
4306 reflections	Δρ_max_ = 0.24 e Å^−^^3^
208 parameters	Δρ_min_ = −0.24 e Å^−^^3^
0 restraints	Absolute structure: Flack (1983), 1853 Friedel pairs
Primary atom site location: structure-invariant direct methods	Flack parameter: 0.03 (7)

### Special details

**Table:**

Geometry. All e.s.d.'s (except the e.s.d. in the dihedral angle between two l.s. planes) are estimated using the full covariance matrix. The cell e.s.d.'s are taken into account individually in the estimation of e.s.d.'s in distances, angles and torsion angles; correlations between e.s.d.'s in cell parameters are only used when they are defined by crystal symmetry. An approximate (isotropic) treatment of cell e.s.d.'s is used for estimating e.s.d.'s involving l.s. planes.
Refinement. Refinement of *F*^2^ against ALL reflections. The weighted *R*-factor *wR* and goodness of fit *S* are based on *F*^2^, conventional *R*-factors *R* are based on *F*, with *F* set to zero for negative *F*^2^. The threshold expression of *F*^2^ > σ(*F*^2^) is used only for calculating *R*-factors(gt) *etc*. and is not relevant to the choice of reflections for refinement. *R*-factors based on *F*^2^ are statistically about twice as large as those based on *F*, and *R*- factors based on ALL data will be even larger.

### Fractional atomic coordinates and isotropic or equivalent isotropic displacement parameters (Å^2^)

**Table:**

	*x*	*y*	*z*	*U*_iso_*/*U*_eq_	
C1	0.3444 (2)	0.27298 (15)	0.91369 (9)	0.0423 (4)	
C2	0.4528 (2)	0.2866 (2)	0.96899 (11)	0.0596 (5)	
H2	0.5302	0.2325	0.9709	0.072*	
C3	0.4447 (3)	0.3813 (2)	1.02106 (13)	0.0737 (7)	
H3	0.5170	0.3910	1.0583	0.088*	
C4	0.3305 (3)	0.4615 (2)	1.01838 (13)	0.0720 (7)	
H4	0.3252	0.5250	1.0539	0.086*	
C5	0.2244 (3)	0.4475 (2)	0.96314 (14)	0.0676 (6)	
H5	0.1478	0.5023	0.9611	0.081*	
C6	0.2295 (2)	0.3532 (2)	0.91061 (11)	0.0542 (5)	
H6	0.1566	0.3438	0.8736	0.065*	
C7	0.61067 (18)	0.20568 (15)	0.77713 (10)	0.0396 (4)	
H7	0.6361	0.1620	0.8249	0.048*	
C8	0.6733 (2)	0.33204 (18)	0.78485 (14)	0.0587 (5)	
H8A	0.6285	0.3732	0.8286	0.070*	
H8B	0.6528	0.3782	0.7381	0.070*	
C9	0.8375 (2)	0.3243 (2)	0.79731 (14)	0.0647 (6)	
H9A	0.8777	0.4054	0.8002	0.078*	
H9B	0.8572	0.2838	0.8463	0.078*	
C10	0.9099 (2)	0.2560 (2)	0.73209 (13)	0.0653 (6)	
H10A	0.8985	0.3008	0.6839	0.078*	
H10B	1.0131	0.2489	0.7428	0.078*	
C11	0.8458 (2)	0.1321 (2)	0.72278 (14)	0.0717 (6)	
H11A	0.8666	0.0845	0.7689	0.086*	
H11B	0.8908	0.0922	0.6787	0.086*	
C12	0.6807 (2)	0.13740 (19)	0.71029 (12)	0.0574 (5)	
H12A	0.6593	0.1780	0.6616	0.069*	
H12B	0.6416	0.0559	0.7077	0.069*	
C13	0.3750 (2)	0.28365 (15)	0.71472 (9)	0.0407 (4)	
H13A	0.4319	0.3571	0.7088	0.049*	
H13B	0.2811	0.3064	0.7358	0.049*	
C14	0.35265 (19)	0.22767 (13)	0.63549 (9)	0.0385 (3)	
C15	0.2610 (2)	0.12964 (17)	0.62532 (12)	0.0556 (5)	
H15	0.2141	0.0962	0.6681	0.067*	
C16	0.2385 (3)	0.0808 (2)	0.55228 (14)	0.0647 (6)	
H16	0.1760	0.0153	0.5463	0.078*	
C17	0.3074 (3)	0.1283 (2)	0.48873 (12)	0.0646 (6)	
H17	0.2917	0.0955	0.4396	0.077*	
C18	0.4001 (3)	0.2249 (2)	0.49795 (12)	0.0668 (6)	
H18	0.4484	0.2568	0.4551	0.080*	
C19	0.4216 (2)	0.27463 (17)	0.57071 (11)	0.0535 (5)	
H19	0.4833	0.3407	0.5762	0.064*	
N1	0.44909 (16)	0.20318 (12)	0.77103 (8)	0.0402 (3)	
O1	0.21214 (15)	0.12969 (13)	0.81853 (8)	0.0590 (4)	
O2	0.43915 (17)	0.05891 (11)	0.88027 (8)	0.0592 (4)	
S1	0.35656 (5)	0.15399 (4)	0.84502 (2)	0.04355 (12)	

### Atomic displacement parameters (Å^2^)

**Table:**

	*U*^11^	*U*^22^	*U*^33^	*U*^12^	*U*^13^	*U*^23^
C1	0.0453 (9)	0.0462 (9)	0.0352 (8)	−0.0024 (8)	0.0063 (8)	0.0056 (7)
C2	0.0564 (12)	0.0720 (13)	0.0504 (11)	0.0075 (11)	−0.0061 (10)	0.0019 (10)
C3	0.0780 (16)	0.0892 (17)	0.0539 (12)	−0.0098 (14)	−0.0089 (12)	−0.0100 (12)
C4	0.0928 (19)	0.0669 (13)	0.0564 (12)	−0.0073 (14)	0.0093 (14)	−0.0126 (11)
C5	0.0761 (15)	0.0617 (13)	0.0651 (14)	0.0124 (12)	0.0139 (12)	−0.0008 (11)
C6	0.0508 (11)	0.0621 (11)	0.0495 (10)	0.0073 (10)	0.0009 (8)	0.0008 (10)
C7	0.0378 (9)	0.0447 (9)	0.0364 (8)	0.0043 (7)	−0.0017 (7)	0.0039 (7)
C8	0.0437 (11)	0.0513 (10)	0.0812 (14)	0.0000 (9)	0.0033 (10)	−0.0147 (10)
C9	0.0432 (11)	0.0695 (13)	0.0815 (14)	−0.0033 (10)	−0.0042 (10)	−0.0146 (12)
C10	0.0351 (10)	0.0957 (17)	0.0651 (13)	0.0074 (11)	−0.0003 (9)	−0.0016 (13)
C11	0.0513 (12)	0.0869 (16)	0.0768 (14)	0.0229 (13)	0.0006 (11)	−0.0247 (13)
C12	0.0501 (11)	0.0611 (11)	0.0610 (12)	0.0101 (9)	−0.0006 (9)	−0.0166 (10)
C13	0.0418 (9)	0.0388 (8)	0.0416 (9)	0.0036 (7)	−0.0034 (7)	0.0009 (7)
C14	0.0357 (8)	0.0376 (7)	0.0423 (8)	0.0045 (7)	−0.0080 (7)	0.0032 (6)
C15	0.0549 (11)	0.0551 (11)	0.0570 (11)	−0.0136 (10)	−0.0014 (9)	0.0008 (9)
C16	0.0671 (14)	0.0558 (11)	0.0712 (14)	−0.0153 (11)	−0.0111 (11)	−0.0097 (11)
C17	0.0855 (16)	0.0613 (12)	0.0469 (11)	0.0000 (11)	−0.0204 (11)	−0.0056 (10)
C18	0.0957 (18)	0.0657 (14)	0.0390 (10)	−0.0094 (13)	−0.0041 (10)	0.0078 (10)
C19	0.0662 (13)	0.0471 (10)	0.0472 (11)	−0.0115 (10)	−0.0069 (9)	0.0067 (9)
N1	0.0387 (8)	0.0455 (8)	0.0365 (7)	0.0043 (6)	−0.0002 (6)	0.0043 (6)
O1	0.0469 (7)	0.0690 (9)	0.0610 (8)	−0.0151 (7)	0.0052 (6)	−0.0039 (7)
O2	0.0711 (9)	0.0436 (7)	0.0629 (8)	0.0059 (7)	0.0071 (7)	0.0168 (6)
S1	0.0449 (2)	0.0415 (2)	0.0442 (2)	−0.0025 (2)	0.0035 (2)	0.00529 (19)

### Geometric parameters (Å, °)

**Table:**

C1—C6	1.380 (3)	C10—H10B	0.9700
C1—C2	1.386 (3)	C11—C12	1.535 (3)
C1—S1	1.7696 (18)	C11—H11A	0.9700
C2—C3	1.378 (3)	C11—H11B	0.9700
C2—H2	0.9300	C12—H12A	0.9700
C3—C4	1.375 (3)	C12—H12B	0.9700
C3—H3	0.9300	C13—N1	1.480 (2)
C4—C5	1.370 (3)	C13—C14	1.510 (2)
C4—H4	0.9300	C13—H13A	0.9700
C5—C6	1.379 (3)	C13—H13B	0.9700
C5—H5	0.9300	C14—C19	1.382 (2)
C6—H6	0.9300	C14—C15	1.383 (2)
C7—N1	1.490 (2)	C15—C16	1.382 (3)
C7—C8	1.515 (3)	C15—H15	0.9300
C7—C12	1.518 (2)	C16—C17	1.368 (3)
C7—H7	0.9800	C16—H16	0.9300
C8—C9	1.528 (3)	C17—C18	1.374 (3)
C8—H8A	0.9700	C17—H17	0.9300
C8—H8B	0.9700	C18—C19	1.380 (3)
C9—C10	1.506 (3)	C18—H18	0.9300
C9—H9A	0.9700	C19—H19	0.9300
C9—H9B	0.9700	N1—S1	1.6240 (14)
C10—C11	1.498 (3)	O1—S1	1.4299 (14)
C10—H10A	0.9700	O2—S1	1.4305 (14)
			
C6—C1—C2	120.51 (18)	C12—C11—H11A	109.3
C6—C1—S1	119.94 (14)	C10—C11—H11B	109.3
C2—C1—S1	119.54 (15)	C12—C11—H11B	109.3
C3—C2—C1	119.3 (2)	H11A—C11—H11B	107.9
C3—C2—H2	120.4	C7—C12—C11	109.44 (16)
C1—C2—H2	120.4	C7—C12—H12A	109.8
C4—C3—C2	120.6 (2)	C11—C12—H12A	109.8
C4—C3—H3	119.7	C7—C12—H12B	109.8
C2—C3—H3	119.7	C11—C12—H12B	109.8
C5—C4—C3	119.6 (2)	H12A—C12—H12B	108.2
C5—C4—H4	120.2	N1—C13—C14	114.01 (13)
C3—C4—H4	120.2	N1—C13—H13A	108.7
C4—C5—C6	121.0 (2)	C14—C13—H13A	108.7
C4—C5—H5	119.5	N1—C13—H13B	108.7
C6—C5—H5	119.5	C14—C13—H13B	108.7
C5—C6—C1	119.05 (19)	H13A—C13—H13B	107.6
C5—C6—H6	120.5	C19—C14—C15	118.11 (16)
C1—C6—H6	120.5	C19—C14—C13	120.72 (15)
N1—C7—C8	113.74 (14)	C15—C14—C13	121.17 (16)
N1—C7—C12	111.15 (15)	C16—C15—C14	120.77 (19)
C8—C7—C12	111.24 (15)	C16—C15—H15	119.6
N1—C7—H7	106.8	C14—C15—H15	119.6
C8—C7—H7	106.8	C17—C16—C15	120.4 (2)
C12—C7—H7	106.8	C17—C16—H16	119.8
C7—C8—C9	109.69 (17)	C15—C16—H16	119.8
C7—C8—H8A	109.7	C16—C17—C18	119.5 (2)
C9—C8—H8A	109.7	C16—C17—H17	120.2
C7—C8—H8B	109.7	C18—C17—H17	120.2
C9—C8—H8B	109.7	C17—C18—C19	120.1 (2)
H8A—C8—H8B	108.2	C17—C18—H18	119.9
C10—C9—C8	111.14 (18)	C19—C18—H18	119.9
C10—C9—H9A	109.4	C18—C19—C14	121.02 (18)
C8—C9—H9A	109.4	C18—C19—H19	119.5
C10—C9—H9B	109.4	C14—C19—H19	119.5
C8—C9—H9B	109.4	C13—N1—C7	119.61 (14)
H9A—C9—H9B	108.0	C13—N1—S1	118.13 (12)
C11—C10—C9	111.26 (19)	C7—N1—S1	118.29 (11)
C11—C10—H10A	109.4	O1—S1—O2	119.39 (9)
C9—C10—H10A	109.4	O1—S1—N1	107.48 (8)
C11—C10—H10B	109.4	O2—S1—N1	107.38 (8)
C9—C10—H10B	109.4	O1—S1—C1	107.04 (9)
H10A—C10—H10B	108.0	O2—S1—C1	107.20 (8)
C10—C11—C12	111.71 (18)	N1—S1—C1	107.90 (7)
C10—C11—H11A	109.3		
			
C6—C1—C2—C3	0.1 (3)	C16—C17—C18—C19	−1.0 (4)
S1—C1—C2—C3	178.36 (17)	C17—C18—C19—C14	1.0 (3)
C1—C2—C3—C4	0.0 (3)	C15—C14—C19—C18	−0.2 (3)
C2—C3—C4—C5	−0.4 (4)	C13—C14—C19—C18	−179.12 (19)
C3—C4—C5—C6	0.7 (4)	C14—C13—N1—C7	90.42 (18)
C4—C5—C6—C1	−0.6 (3)	C14—C13—N1—S1	−112.35 (15)
C2—C1—C6—C5	0.3 (3)	C8—C7—N1—C13	49.3 (2)
S1—C1—C6—C5	−178.03 (16)	C12—C7—N1—C13	−77.18 (18)
N1—C7—C8—C9	175.43 (16)	C8—C7—N1—S1	−107.91 (16)
C12—C7—C8—C9	−58.1 (2)	C12—C7—N1—S1	125.63 (14)
C7—C8—C9—C10	56.9 (2)	C13—N1—S1—O1	37.74 (14)
C8—C9—C10—C11	−56.2 (3)	C7—N1—S1—O1	−164.73 (12)
C9—C10—C11—C12	55.8 (3)	C13—N1—S1—O2	167.36 (12)
N1—C7—C12—C11	−174.82 (17)	C7—N1—S1—O2	−35.11 (15)
C8—C7—C12—C11	57.3 (2)	C13—N1—S1—C1	−77.38 (14)
C10—C11—C12—C7	−56.0 (3)	C7—N1—S1—C1	80.15 (14)
N1—C13—C14—C19	−115.08 (18)	C6—C1—S1—O1	−25.17 (17)
N1—C13—C14—C15	66.1 (2)	C2—C1—S1—O1	156.51 (15)
C19—C14—C15—C16	−0.5 (3)	C6—C1—S1—O2	−154.38 (14)
C13—C14—C15—C16	178.40 (19)	C2—C1—S1—O2	27.30 (17)
C14—C15—C16—C17	0.5 (3)	C6—C1—S1—N1	90.25 (16)
C15—C16—C17—C18	0.3 (4)	C2—C1—S1—N1	−88.07 (16)

### Hydrogen-bond geometry (Å, °)

**Table:**

*D*—H···*A*	*D*—H	H···*A*	*D*···*A*	*D*—H···*A*
C7—H7···O2	0.98	2.34	2.874 (2)	113
